# Loss of α-Synuclein Does Not Affect Mitochondrial Bioenergetics in Rodent Neurons

**DOI:** 10.1523/ENEURO.0216-16.2017

**Published:** 2017-04-28

**Authors:** Divya Pathak, Amandine Berthet, Jacob T. Bendor, Katharine Yu, Rhyomi C. Sellnow, Adam L. Orr, Mai K Nguyen, Robert H. Edwards, Fredric P. Manfredsson, Ken Nakamura

**Affiliations:** 1 Gladstone Institute of Neurological Disease, San Francisco, CA 94158; 2 Department of Neurology and Graduate Programs in Neuroscience and Biomedical Sciences, University of California, San Francisco, San Francisco, California 94158; 3 Department of Translational Science & Molecular Medicine, College of Human Medicine, Michigan State University, Grand Rapids, MI 49503; 4 Mercy Health Hauenstein Neuroscience Center, Grand Rapids, MI 49503

**Keywords:** bioenergetics, Mitochondria, Neurodegeneration, Parkinson’s Disease, Synuclein

## Abstract

Increased α-synuclein (αsyn) and mitochondrial dysfunction play central roles in the pathogenesis of Parkinson’s disease (PD), and lowering αsyn is under intensive investigation as a therapeutic strategy for PD. Increased αsyn levels disrupt mitochondria and impair respiration, while reduced αsyn protects against mitochondrial toxins, suggesting that interactions between αsyn and mitochondria influences the pathologic and physiologic functions of αsyn. However, we do not know if αsyn affects normal mitochondrial function or if lowering αsyn levels impacts bioenergetic function, especially at the nerve terminal where αsyn is enriched. To determine if αsyn is required for normal mitochondrial function in neurons, we comprehensively evaluated how lowering αsyn affects mitochondrial function. We found that αsyn knockout (KO) does not affect the respiration of cultured hippocampal neurons or cortical and dopaminergic synaptosomes, and that neither loss of αsyn nor all three (α, β and γ) syn isoforms decreased mitochondria-derived ATP levels at the synapse. Similarly, neither αsyn KO nor knockdown altered the capacity of synaptic mitochondria to meet the energy requirements of synaptic vesicle cycling or influenced the localization of mitochondria to dopamine (DA) synapses *in vivo*. Finally, αsyn KO did not affect overall energy metabolism in mice assessed with a Comprehensive Lab Animal Monitoring System. These studies suggest either that αsyn has little or no significant physiological effect on mitochondrial bioenergetic function, or that any such functions are fully compensated for when lost. These results implicate that αsyn levels can be reduced in neurons without impairing (or improving) mitochondrial bioenergetics or distribution.

## Significance Statement

Parkinson’s disease (PD) is characterized by mitochondrial dysfunction and the accumulation of α-synuclein (αsyn), and lowering αsyn levels is a leading therapeutic strategy for PD that is already under clinical investigation. However, because αsyn and mitochondria have intersecting functions, we must understand the impact of lowering αsyn on mitochondrial function. We analyzed the effects of lowering αsyn on mitochondrial bioenergetics, particularly at the nerve terminal where αsyn concentrates. We found that loss of αsyn does not impact the intrinsic bioenergetic function of mitochondria, suggesting that αsyn does not normally influence respiration, and that αsyn levels can likely be lowered without affecting mitochondrial function in PD.

## Introduction

Increased α-synuclein (αsyn) and mitochondrial dysfunction contribute to the pathogenesis of Parkinson’s disease (PD). For example, mutation or overexpression of wild-type αsyn and mitochondrial or mitochondria-associated proteins (e.g., PINK1, Parkin, and CHCHD2) cause monogenic forms of PD (Polymeropoulos et al. 1997; Kruger et al. 1998; Singleton et al. 2003; Valente et al. 2004; Zarranz et al. 2004; Clark et al. 2006; Park et al. 2006; Funayama et al. 2015). In sporadic PD, αsyn accumulates in Lewy bodies and dystrophic neurites (Spillantini et al. 1998), and mitochondria exhibit prominent changes, including decreased complex I activity in the substantia nigra (SN) (Schapira et al. 1990) and increased accumulation of mutations in the mitochondrial DNA of surviving SN dopamine (DA) neurons (Bender et al. 2006). In addition, changes occur in genes that regulate mitochondrial functions, including reduced expression of peroxisome proliferator-activated receptor-γ coactivator (PGC)-1α and PGC1α-regulated genes in SN dopamine (DA) neurons (Zheng et al. 2010).

αSyn and mitochondria also directly affect each other. In mice and humans, a fraction of αsyn associates with mitochondria in DA neurons (Martin et al. 2006; Li et al. 2007; Devi et al. 2008). Additionally, supra-physiologic levels of αsyn disrupt mitochondrial morphology *in vitro* and *in vivo* (Kamp et al. 2010; Nakamura et al. 2011; Butler et al. 2012), promote excessive mitophagy (Choubey et al. 2011; Sampaio-Marques et al. 2012), disrupt mitochondrial protein import (Di Maio et al. 2016) and influence mitochondrial Ca^2+^ homeostasis and apposition between the endoplasmic reticulum and mitochondria (Cali et al. 2012; Guardia-Laguarta et al. 2014). Increased αsyn also inhibits mitochondrial complex I in cell lines and mouse brains (Devi et al. 2008; Liu et al. 2009; Chinta et al. 2010; Loeb et al. 2010), and it correlates with decreased complex I activity in mitochondrial fractions from the SN of PD patients (Devi et al. 2008).

The effects of increased αsyn on mitochondria may result from a toxic gain-of-function, such as the accumulation of oligomeric αsyn species that interact preferentially with mitochondria (van Rooijen et al. 2009; Nakamura et al. 2011; Nakamura, 2013; Luth et al. 2014). Moreover, even basal levels of αsyn may predispose mitochondria to dysfunction. In αsyn knockout (KO) mice, SN DA neurons resist toxicity from mitochondrial toxins, including 1-methyl-4-phenyl-1,2,3,6-tetrahydropyridine (MPTP) (Dauer et al. 2002; Klivenyi et al. 2006), and reducing endogenous αsyn protects against toxicity from the complex I inhibitor rotenone (Zharikov et al. 2015) and may improve mitochondrial protein import (Di Maio et al. 2016). However, excessively lowering αsyn could also adversely affect mitochondria. While increased αsyn causes mitochondrial fragmentation, decreased αsyn can produce excessive mitochondrial tubulation (Kamp et al. 2010; Norris et al. 2015). Additionally, αsyn KO mice have decreased levels of the mitochondrial lipid cardiolipin (Ellis et al. 2005) and lowering αsyn impairs complex I/III activity, perhaps by directly interacting with complex I (Ellis et al. 2005; Devi et al. 2008). In addition, syn TKO mitochondria have decreased mitochondrial membrane potential but increased oxygen consumption, and lower activity of ATP synthase (Ludtmann et al. 2016).

Both αsyn and mitochondria are major targets for PD therapy. Lowering αsyn is under study in clinical trials (Dehay et al. 2015), but whether this approach is safe remains unknown. Interestingly, although single-syn KO (α, β, or γ) and double-syn KO mice (α and β) have normal lifespans (Chandra et al. 2004), triple-syn KO mice (α, β, and γ) die early (Greten-Harrison et al. 2010). These results suggest that αsyn mediates essential functions, but other syn isoforms can compensate for its loss, at least over the lifespan of a mouse. In addition, the capacity to compensate for αsyn loss may decrease once neuronal maturation is complete. Indeed, some studies found that lowering αsyn in adult rodents (Benskey et al. 2016b) or non-human primates (Collier et al. 2016) using shRNA is toxic to nigral DA neurons, while other studies have not (McCormack et al. 2010; Zharikov et al. 2015), with the discrepancies likely due to varying degrees of αsyn knockdown. Therefore, we must better understand the consequences of lowering αsyn, particularly on mitochondrial function in axons where αsyn concentrates and energy failure can selectively occur (Shields et al. 2015).

In this study, we aimed to determine if αsyn is required for normal mitochondrial bioenergetics, and if αsyn levels can be safely lowered without affecting mitochondrial bioenergetics. We comprehensively evaluated how the loss of αsyn impacts mitochondrial function, including respiration in neurons and isolated nerve terminals, mitochondrial-derived ATP levels specifically at synapses of intact neurons, localization of mitochondria to DA synapses *in vivo*, and total bioenergetic function.

## Materials and Methods

### Molecular Biology

All constructs used for transient transfection were subcloned or cloned into the pCAGGS vector downstream of the chicken actin promoter (Voglmaier et al. 2006). The AT1.03^YEMK^ FRET sensor was a kind gift from Dr Hiroyuki Noji at Osaka University (Imamura et al. 2009a). VGLUT1-pHluorin-mCherry is derived from the VGLUT1-pHluorin fusion (Voglmaier et al. 2006) and was a kind gift from Dr Timothy Ryan (Weil Cornell Medical School), mCherry-synaptophysin has been described (Voglmaier et al. 2006; Hua et al. 2011), and mTagBFP was a kind gift from Dr Vladislav Verkhusha at the Albert Einstein College of Medicine (Subach et al. 2008). Mitochondria-targeted GFP (mitoGFP) and mCherry fused to the N-terminus of rat synaptophysin (Hua et al. 2011) were subcloned into pAAV-EF1a-DIO-hChR2(H134R)-EYFP-WPRE (Addgene), and recombinant adeno-associated virus (AAV)1 was made by the Vector Core at the University of North Carolina.

Viral constructs for αsyn silencing were similar to those used before (Gorbatyuk et al. 2010); the siRNA sequences [αsyn: GAAGGACCAGATGGGCAAG, scrambled (SCR): GTCGACAATTCATATTTGC] were expressed as a shRNA by incorporating the loop structure TTCAAGAGA. The shRNA cassette was inserted behind an H1 promoter, and the viral genome also contained mTagBFP2 under the control of the hybrid chicken β-actin/cytomegalovirus enhancer promoter (pCBA) as a transduction marker. The viral vectors were packaged into AAV5 capsids by transfection of 293 cells with the viral genome and the pXYZ5 helper plasmid. Viral particles were purified using an iodixanol gradient followed by column chromatography, and titers were determined by dot-blot (Benskey et al. 2016a).

### Knockout and Transgenic Mice

αSyn KO mice on a C57BL/6N background (strain 016123, The Jackson Laboratory) (Baptista et al. 2013) were used for most experiments. Before these mice were available, αsyn KO mice on a mixed C57BL/6 and 129 × 1/SvJ background (strain 003692, The Jackson Laboratory, backcrossed one generation with C57BL/6N controls) were used for experiments outlined in Fig. 2B. DATcre (Backman et al. 2006) mice were also obtained from The Jackson Laboratory. C57BL/6N mice served as controls for all studies. Mice were group-housed in a colony maintained with a standard 12-h light/dark cycle and given food on the cage floor and water ad libitum. All experiments were performed on age- and sex-matched mice. Experiments were conducted in accordance with the *Guide for the Care and Use of Laboratory Animals*, as adopted by the National Institutes of Health and with approval of the Authors’ University Institutional Animal Care and Use Committee.

### Cell Culture

Postnatal hippocampal neurons for live-imaging experiments were prepared from P0 rat (Sprague Dawley) or mouse pups. Hippocampi were dissected in 37˚C Hanks’ BSS supplemented with glucose (20 mM) and HEPES (10 mM) (HBSS++) and plated at a density of 85 cells/mm^2^. Neurons are grown in neuronal media consisting of Earle’s Minimum Essential Medium supplemented with 5% fetal bovine serum (FBS), 21 mM glucose, 1% Glutamax, 2% B27 supplement (Gibco 17504-044), and 0.1% serum extender (Fisher 355006). Cells for imaging were transiently transfected by electroporation (Amaxa) and cultured for 8–14 days before live imaging or analysis (Fig. 2), or transfected with Ca^2+^-phosphate (Jiang and Chen, 2006) on DIV8 and cultured 14–20 days before imaging (Fig. 3). Hippocampal neurons for Seahorse experiments were prepared from E18 embryos (to minimize glial contamination (Yao et al. 2011)), plated at a density of 5 × 10^4^ cells per well of a 96-well polystyrene microplate, and cultured for 10–11 days before analysis.

### Synaptosome Isolation

Cortical synaptosomes were isolated from cerebral cortices of 6-month-old mice as described (Gerencser et al. 2009). Briefly, cortices were quickly dissected, rinsed, and gently homogenized in ice-cold sucrose buffer (320 mM sucrose, 1 mM EDTA, 0.25 mM dithiothreitol, pH 7.4). Homogenates were centrifuged at 1000 × *g* for 10 min at 4°C. The supernatant was layered on top of a discontinuous Percoll gradient of 3, 10, and 23% Percoll layers in sucrose medium and centrifuged at 32500 × *g* for 10 min at 4°C. Synaptosomes accumulated as a band between the 10% and 23% Percoll layers and were gently aspirated and washed in HBS medium containing 20 mM HEPES, 10 mM D-glucose, 1.2 mM Na_2_HPO_4_, 1 mM MgCl_2_, 5 mM NaHCO_3_, 5 mM KCl, and 140 mM NaCl at pH. 7.4. The final synaptosomal pellet was resuspended in HBS medium.

To prepare dopaminergic synaptosomes, mice striata were quickly dissected and homogenized using ice-cold sucrose buffer (Choi et al. 2011). Homogenates were then incubated with antibodies against the dopamine transporter (Alpha Diagnostic International; 25 *µ*g/sample) for 60 min at 4°C and washed three times in sucrose buffer at 10 000 × *g* for 2 min. Pellets were then incubated with 150 *μ*l of secondary IgG magnetic beads (Miltenyi) for 45 min at 4°C and then poured into a magnetic column (MACS LS; Miltenyi) to separate the magnetic bead–labeled dopaminergic synaptosomes (bound to column, DA) from the nondopaminergic fraction that flows through the column.

### Western Blot Analysis

Relative levels of αsyn (1:2000; catalog #610787, BD Biosciences, RRID:AB_398108), tyrosine hydroxylase (1:4000; catalog #MAB318, EMD Millipore, RRID:AB_2201528), and synaptophysin (1:1000; catalog #ab68851, Abcam, RID:AB_2199023) in dopaminergic and non-dopaminergic synaptosomes were determined by western blotting by standard procedures. Relative levels of synuclein isoforms [αsyn (1:1000; catalog #610787, BD Biosciences, RRID:AB_398108); βsyn (1:1000; catalog #AB5086, EMD Millipore Chemicals, RRID:AB_2239676)] in brain lysates were also assessed by western blotting. Samples of synaptosomes (23 *µ*g) or brain lysates (25 *µ*g) were denatured by boiling in an equal volume of 2× Laemmli Lysis Buffer (Bio-Rad) for 5 min. Proteins were resolved by SDS-PAGE before transfer to PVDF membranes and detected with Pierce ECL substrate (ThermoFisher).

### Respiration and Glycolysis

The extracellular-acidification rates (ECAR, a surrogate for glycolysis) and oxygen-consumption rates (OCR, assesses mitochondrial respiration) were measured in cultured hippocampal neurons using a Seahorse XF96 Extracellular Flux Analyzer (Seahorse Bioscience), an instrument that can simultaneously assess aerobic and anaerobic metabolism in adherent cells cultured in 96-well plates. Cells were washed and preincubated for 30 min in Seahorse assay medium (pH 7.4) containing substrates of interest (30 mM glucose and 10 mM pyruvate). OCR and ECAR were measured at baseline and again after sequential addition of the respiratory inhibitors FCCP (1 µM), rotenone (3 µM), and oligomycin (2 µM), or with veratridine (50 µM) to increase neural activity (Lysko et al. 1994). To assess any effects of genotype on cell survival, cells in a subset of wells were also stained with DAPI (4',6-diamidino-2-phenylindole) and quantified using MetaMorph software (Universal Imaging).

Aerobic respiration was also measured in cortical and dopaminergic synaptosomes prepared from αsyn KO and control mice. Before plating synaptosomes, Seahorse plates were coated with 0.0033% (v/v) polyethyleneimine solution and Geltrex suspension. Synaptosomes were then added to each well (cortical 20 *μ*g/well; dopaminergic 40 *μg*/well), and the plates were centrifuged at 3200 × *g* for 50 mins at 4°C to attach the synaptosomes to the surface. For Seahorse measurements, HBS medium was replaced with Seahorse buffer containing 3.5 mM KCl, 120 mM NaCl, 1.3 mM CaCl_2,_ 0.4 mM KH_2_ PO_4_, 1.2 mM Na_2_SO_4_, 2 mM MgSO_4_, 10 mM TES, 10 mM Na-pyruvate, and 4 mg/ml bovine serum albumin.

### Neuronal Culture and Live Imaging

Live imaging was performed in Tyrode’s medium (pH 7.4; 127 mM NaCl, 10 mM HEPES-NaOH, 2.5 mM KCl, 2 mM MgCl_2_, 2 mM CaCl_2_, and 10 mM pyruvate, with or without 30 mM glucose) at room temperature (Fig. 2) on a Nikon Ti-E inverted microscope with an iXon EMCCD camera (Andor Technology) and a perfusion valve–control system (VC-8, Warner Instruments) or at 35°C (Fig. 3) on a Nikon Eclipse TE300 inverted microscope with a QuantEM:512SC EMCCD camera (Photometrics) controlled by MetaMorph Software. Field stimulation (5–30 Hz) was performed with an A385 current isolator and a SYS-A310 accupulser–signal generator (World Precision Instruments). Glycolysis was inhibited with 2-deoxyglucose (2DG, 2.5–5 mM; Sigma-Aldrich) and iodoacetate (1 mM; Sigma-Aldrich).

VGLUT1-pHluorin fluorescence images were obtained [490/20 excitation (ex), 535/50 emission (em) (Fig. 2) or 470/40 ex, 525/50 em (Fig 3); Chroma] and regions of interest were drawn over synaptic boutons, identified based on co-localization with mCherry-synaptophysin (Fig. 2) or VGLUT1-pHluorin-mCherry (Fig. 3). The background-subtracted change in fluorescence at each time point was then normalized to the fluorescence in ammonium chloride measured at the end of each run (Fig. 2) (Nemani et al. 2010) or to the peak fluorescence response (ΔF) to the initial stimulus train (Fig. 3). For fluorescence resonance energy transfer (FRET) experiments, sequential images were taken in the CFP [430/24 ex, 470/24 em (Fig. 2) or 436/10 ex, 465/30 em (Fig.3)], YFP [(500/20 ex, 535/30 em (Fig. 2) or 495/10 ex, 525/30 em (Fig. 3)], and FRET [(430/24 ex, 535/30 em (Fig. 2) or 436/10 ex, 525/30 em (Fig. 3)] channels with an ET ECFP/EYFP filter set (Chroma). Synaptic boutons were identified based on co-localization with mCherry-synaptophysin. The FRET/donor ratio was calculated for each bouton as described (Xia and Liu, 2001), where FRET = (I_FRET_ − I_CFP_ * BT_CFP_ – I_YFP_ * BT_YFP_)/I_CFP_, such that *I_X_* is the background-corrected fluorescence intensity measured in a given channel. BT_CFP_ (donor bleed through) and BT_YFP_ (direct excitation of the acceptor) were calculated by expressing CFP and YFP individually and then determining the ratios of I_FRET_/I_CFP_ and I_FRET_/I_YFP_, respectively.

### Stereotaxic Recombinant Adeno-Associated Virus and Injection

Stereotaxic injections of adeno-associated virus 1 (AAV1) expressing mitoGFP and mCherry-Synaptophysin were performed in 3- and 7-month-old Dat^icre/wt^ and Dat^icre/wt^ KO mice. Briefly, 0.5 *μ*l of AAV1-EF1α-DIO-mitoGFP (cre-dependent mitoGFP, 8 × 10^12^ VG/ml) and 0.5 *μ*l of AAV1-EF1α-DIO-Cherry-synaptophysin (cre-dependent Cherry-synaptophysin, 3 × 10^12^ VG/ml) were co-injected unilaterally into the substantia nigra/VTA (anteroposterior, –3.0 mm from bregma; mediolateral, 1.1 mm; dorsoventral, 4.3 mm). Animals were sacrificed 4 weeks after injection. Seven-month-old Dat^icre/wt^ mice were co-injected with AAV1 expressing cre-dependent mitoGFP and/or cre-dependent mCherrySynaptophysin, as well as 0.5 *μ*l of either AAV2/5-αsyn-shRNA-mTagBFP2 (1.8 × 10^13^ VG/ml) or AAV2/5- SCR-shRNA-mTagTagBFP (1.1 × 10^13^ VG/ml), and sacrificed 6 weeks after injection.

## Histology

Mice were anesthetized and perfused with phosphate buffered saline (PBS) and then 4% paraformaldehyde (PFA). Brains were sectioned and processed for immunofluorescence. The following primary and secondary antibodies were used: anti–tyrosine hydroxylase (TH) [(mouse, 1:20000; catalog #MAB318, EMD Millipore, RRID:AB_2201528) and (rabbit, 1:1000; catalog #AB152, Millipore, RRID:AB_390204)], rabbit anti-DsRed (1:1000; catalog #632496, Clontech, RRID:AB_10015246), mouse anti-αsyn (1:400; catalog #610787, BD Biosciences, RRID:AB_2201528), and Alexa Fluor 488, 594, or 647 anti-mouse or -rabbit IgG (1:300; Invitrogen). Samples were imaged with the examiner blind to the genotype with a laser-scanning confocal microscope (LSM510-Meta; Carl Zeiss) equipped with 63 × *(*1.4 NA) and 100 × *(*1.3 NA) PlanApo objectives. For a given experiment, all images were captured using the same excitation and emission settings.

## Stereology

Unbiased stereology was used to quantify the number of TH-positive neurons in the SN as described (Gorbatyuk et al. 2010). Sections were visualized using 4x magnification (Olympus BX53 microscope equipped with a motorized stage (Olympus, Center Valley, PA) and a Qimaging 2000R camera (Qimaging, Surrey, BC, Canada)), and the SN was outlined. TH+ cells from every sixth section were counted using the optical fractionator method with a 60x oil objective (Stereo Investigator, MBF Bioscience). The coefficient of error was calculated according to Gundersen and Jensen (Gundersen and Jensen, 1987) and was <0.1 (Gundersen, m = 1).

## Comprehensive Lab Animal Monitoring System Measurements

For metabolic measurements, a Comprehensive Lab Animal Monitoring System (CLAMS, Columbus Instruments) was used to measure the rates of O_2_ (VO_2_) and CO_2_ consumption (VCO_2_), the respiratory-exchange ratio (RER; [dot]Vco_2_/VO_2_), and the activity level of 6-month-old male mice (Millership et al. 2012). These measurements were calculated for both the dark and light cycle for 3 consecutive days. The animals were maintained on a regular chow diet (10% kcal from fat). The body composition (lean and fat mass) of the control and syn KO mice was also analyzed using EchoMRI (Yu et al. 2013).

## Results

### Loss of αsyn Does Not Affect Mitochondrial Bioenergetics at the Nerve Terminal

**Figure 1.**
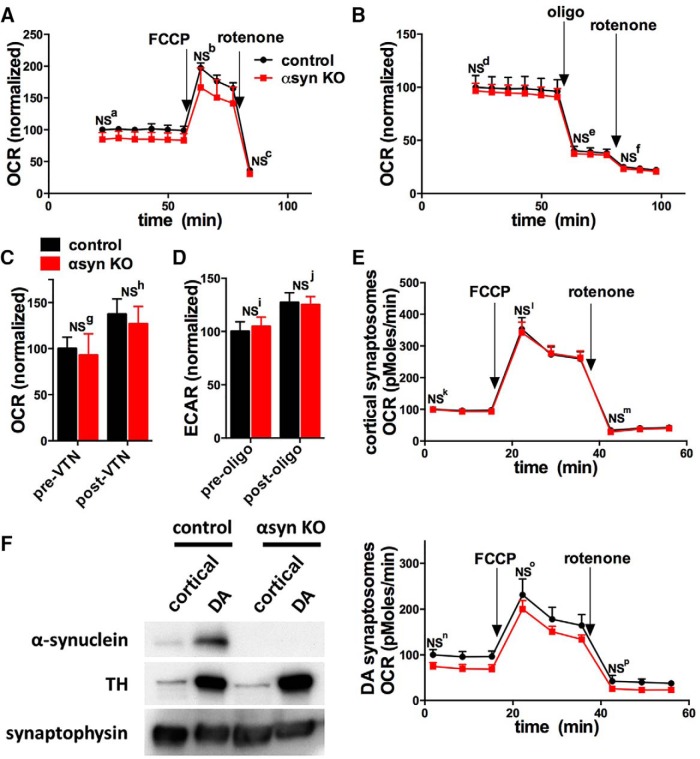
αSyn KO does not impact respiration in cultured neurons or synaptosomes. Aerobic respiration rates (oxygen consumption rate, OCR) were measured using a 96-well Seahorse Extracellular Flux Analyzer. Arrows show addition of the mitochondrial uncoupler FCCP (1 *µ*M for neurons; 3 *µ*M for synaptosomes), the ATP synthase inhibitor oligomycin (oligo, 2 *µ*M), or the mitochondrial complex I inhibitor rotenone (3 *µ*M for neurons and synaptosomes). ***A***, αSyn KO had no effect on the basal or maximal (after FCCP) respiration of hippocampal neurons in medium containing 10 mM pyruvate and 30 mM glucose (compilation of two experiments, *n* = 13 wells per group). ***B***, Oligomycin and rotenone similarly decrease OCR in αsyn KO and control groups (compilation of two experiments, *n* = 10 wells per group). ***C***, Increasing neuronal activity with veratridine similarly increased OCR (compilation of two experiments, *n* = 6 wells per group), while oligomycin similarly increased ECAR (***D***; extracellular acidification rate, a surrogate of glycolysis; compilation of two experiments, *n* = 9 wells per group). ***E***, ***F***, cortical synaptosomes (***E***) and dopamine (DA) synaptosomes (***F*, *right***) isolated from the striatum also had similar basal and maximal rates of respiration (*n* = 15 wells per group from two experiments for cortical synaptosomes; *n* = 7–8 wells per group from two experiments for DA synaptosomes). As expected, western blotting (***F*, *left***) shows that both control and αsyn KO DA synaptosomes are enriched in tyrosine hydroxylase (TH). All graphs show mean ± SEM. NS = not significant by two-way ANOVA and Sidak’s posthoc test.

To determine if αsyn is required for neuronal respiration, we measured how αsyn KO affects the oxygen consumption rate (OCR, a surrogate of respiration) of E18 hippocampal neuronal cultures with a Seahorse instrument. We examined hippocampal neurons because αsyn biology has been extensively characterized in hippocampal neurons in culture and *in vivo* (Greten-Harrison et al. 2010; Nemani et al. 2010; Scott et al. 2010; Volpicelli-Daley et al. 2011), and αsyn also aggregates in hippocampal neurons in PD (Hall et al. 2014). In addition, in contrast to dopamine (DA) neurons that constitute only a fraction of the total neurons in midbrain cultures, hippocampal neuronal cultures consist primarily of excitatory pyramidal neurons (Beaudoin et al. 2012), facilitating their analysis in bulk assays, such as with the Seahorse. Neuronal cultures were grown in serum-free media to minimize the glial content so that respiration is responsible for most of the OCR signal (Pathak et al. 2015). We found that αsyn KO cultures had similar basal and maximal (after treatment with 1 *µ*M FCCP) respiration as controls (Fig. 1*A*, *B*). In addition, increasing neuronal activity with veratridine (50 *µ*M) augmented OCR similarly in both groups (Fig. 1*C*), suggesting that the respiratory function of αsyn KO neurons upregulates normally when energy requirements are increased. Nonetheless, there was a small trend for αsyn KO to have decreased basal OCR in several runs (Figs. 1*A*–*C*), and we cannot exclude the possibility that αsyn KO causes a small impairment in respiration that was below the sensitivity of the assay (see statistical table). Notably, αsyn KO neurons also had similar basal rates of extracellular acidification (ECAR, a surrogate of glycolysis), which increased similarly after treatment with oligomycin (2 *µ*M) (Fig. 1*D*). These results suggest that αsyn KO neurons also have normal glycolytic capacity. Notably αsyn KO also did not affect the total number of surviving cells per well, which was assessed in a subset of wells by DAPI staining (control: 296.9 cells/100 mm^2^**±** 6.0, αsyn KO: 305.7 cells/100 mm^2^**±** 7.3; mean **±** SE, 30 wells per group).

**Table 1.** Statistics

**Row**	**Figure**	**Data Distribution**	**Test**	**Power for 10% change**	**Power for 25% change**	**Power for 50% change**	**Value detected with β 0.1, α 0.05**
**a**	1*A*, Seahorse-neuron-baseline	Normal	Two-way *ANOVA*, Sidak’s *post hoc* test	0.54	1	1	84.2
**b**	1*A*, Seahorse-neuron-FCCP	Normal	Two-way *ANOVA*, Sidak’s *post hoc* test	0.69	1	1	171
**c**	1*A*, Seahorse-neuron-rotenone	Normal	Two-way *ANOVA*, Sidak’s *post hoc* test	0.33	0.97	1	28.6
**d**	1*B*, Seahorse-neuron-baseline	Normal	Two-way *ANOVA*, Sidak’s *post hoc* test	0.15	0.62	1	64.5
**e**	1*B*, Seahorse neuron-oligomycin	Normal	Two-way *ANOVA*, Sidak’s *post hoc* test	0.16	0.68	1	26.8
**f**	1*B*, Seahorse neuron-rotenone	Normal	Two-way *ANOVA*, Sidak’s *post hoc* test	0.17	0.71	1	16.8
**g**	1*C*, Seahorse neuron pre- veratridine	Normal	Two-way *ANOVA*, Sidak’s *post hoc* test	0.13	0.53	0.98	60
**h**	1*C*, Seahorse neuron post- veratridine	Normal	Two-way *ANOVA*, Sidak’s *post hoc* test	0.13	0.54	0.98	83
**i**	1*D*, Seahorse neuron pre- oligomycin	Normal	Two-way *ANOVA*, Sidak’s *post hoc* test	0.2	0.79	1	70.5
**j**	1*D*, Seahorse neuron post- oligomycin	Normal	Two-way *ANOVA*, Sidak’s *post hoc* test	0.28	0.93	1	97.5
**k**	1*E*, Seahorse, cortical synaptosomes-baseline	Normal	Two-way *ANOVA*, Sidak’s *post hoc* test	0.31	0.95	1	77.5
**l**	1*E*, Seahorse, cortical synaptosomes-FCCP	Normal	Two-way *ANOVA*, Sidak’s *post hoc* test	0.16	0.68	1	235
**m**	1*E*, cortical synaptosomes- rotenone	Normal	Two-way *ANOVA*, Sidak’s *post hoc* test	0.08	0.3	0.82	15
**n**	1*F*, DA synaptosomes-baseline	Normal	Two-way *ANOVA*, Sidak’s *post hoc* test	0.14	0.6	0.99	63.5
**o**	1*F*, DA synaptosomes-FCCP	Normal	Two-way *ANOVA*, Sidak’s *post hoc* test	0.1	0.39	0.92	120
**p**	1*F*, DA synaptosomes-rotenone	Normal	Two-way *ANOVA*, Sidak’s *post hoc* test	0.05	0.13	0.37	0
**q**	2*A*, baseline ATP sensor	Normal	Unpaired Student’s *t-*test (two-tailed)	0.23	0.87	1	2
**r**	2*B*, poststim1-imagea	Normal	Two-way *ANOVA*, Sidak’s *post hoc* test	0.97	1	1	77.2
**s**	2*B*, poststim1-Imageb	Normal	Two-way *ANOVA*, Sidak’s *post hoc* test	0.56	1	1	74.8
**t**	2*B*, poststim2-imagea	Normal	Two-way *ANOVA*, Sidak’s *post hoc* test	0.41	0.99	1	66
**u**	2*B*, poststim2-imageb	Normal	Two-way *ANOVA*, Sidak’s *post hoc* test	0.75	1	1	70.5
**v**	2*C*, poststim1-imagea	Normal	Two-way *ANOVA*, Sidak’s *post hoc* test	0.89	1	1	74.6
**w**	2*C*, poststim1-Imageb	Normal	Two-way *ANOVA*, Sidak’s *post hoc* test	0.99	1	1	85.1
**x**	2*C*, poststim2-imagea	Normal	Two-way *ANOVA*, Sidak’s *post hoc* test	0.82	1	1	65.9
**y**	2*C*, poststim2-imageb	Normal	Two-way *ANOVA*, Sidak’s *post hoc* test	0.93	1	1	63.8
**z**	2*D*, synKO pHluorin-stim1	Normal	Two-way *ANOVA*, Sidak’s *post hoc* test	0.51	1	1	0.794
**aa**	2*D*, synKO pHluorin-stim2	Normal	Two-way *ANOVA*, Sidak’s *post hoc* test	0.3	0.95	1	0.718
**ab**	2*E*, syn knockdown level	Normal	Unpaired Student’s *t*-test (two-tailed)	0.28	0.93	1	76.5
**ac**	2*F*, syn shRNA pHluorin-stim 1	Normal	Two-way *ANOVA*, Sidak’s *post hoc* test	0.07	0.23	0.7	0.38
**ad**	2*F*, syn shRNA pHluorin-stim 2	Normal	Two-way *ANOVA*, Sidak’s *post hoc* test	0.85	1	1	0.932
**ae**	3*A*, syn TKO ATP sensor	Normal	Two-way *ANOVA*, Sidak’s *post hoc* test	0.21	0.82	1	0.645
**af**	3*D*, syn TKO pHluorin	Normal	Unpaired Student’s *t*-test (two-tailed)	0.32	0.96	1	2.93
**ag**	4*B*, syn KO mito boutons 4 months	Normal	Unpaired Student’s *t*-test (two-tailed)	0.42	0.99	1	47.5
**ah**	4*B*, syn KO mito boutons 8 months	Normal	Unpaired Student’s *t*-test (two-tailed)	1	1	1	54.1
**ai**	4*C*, Western αsyn	Normal	One-way *ANOVA*, Dunnett’s *post hoc* test	0.6	1	1	85.2
**aj**	4*C*, Western βsyn	Normal	One-way *ANOVA*, Dunnett’s *post hoc* test	0.05	0.13	0.37	1
**ak**	4*E*, shRNA syn level *in vivo*	Normal	Unpaired Student’s *t*-test (two-tailed)	1	1	1	75.4
**al**	4*F*, stereology	Normal	Unpaired Student’s *t*-test (two-tailed)	0.47	1	1	0.715
**am**	4*G*, shRNA mito boutons	Normal	Unpaired Student’s *t*-test (two-tailed)	0.72	1	1	50.5
**an**	5*A*, lean body mass	Normal	Two-way *ANOVA*, Sidak’s *post hoc* test	1	1	1	20.1
**ao**	5*A*, fat body mass	Normal	Two-way *ANOVA*, Sidak’s *post hoc* test	0.39	0.99	1	2.58
**ap**	5*B*, food intake	Normal	Unpaired Student’s *t*-test (two-tailed)	0.42	0.99	1	4
**aq**	5*C*, activity light cycle	Normal	Two-way *ANOVA*, Sidak’s *post hoc* test	0.06	0.18	0.54	53
**ar**	5*C*, activity dark cycle	Normal	Two-way *ANOVA*, Sidak’s *post hoc* test	0.09	0.36	0.9	360
**as**	5*D*, VO_2_ light cycle	Normal	Two-way *ANOVA*, Sidak’s *post hoc* test	1	1	1	2.99
**at**	5*D*, VO_2_ dark cycle	Normal	Two-way *ANOVA*, Sidak’s *post hoc* test	1	1	1	3.64
**au**	5*E*, VCO_2_ light cycle	Normal	Two-way *ANOVA*, Sidak’s *post hoc* test	0.1	0.4	0.93	1.68
**av**	5*E*, VCO_2_ dark cycle	Normal	Two-way *ANOVA*, Sidak’s *post hoc* test	0.1	0.4	0.93	1.68
**aw**	5*F*, RER light cycle	Normal	Two-way *ANOVA*, Sidak’s *post hoc* test	1	1	1	0.829
**ax**	5*F*, RER dark cycle	Normal	Two-way *ANOVA*, Sidak’s *post hoc* test	1	1	1	0.853

The aforementioned measurements interrogate the overall respiration of neurons, but they may not be sensitive to changes in specific subcellular compartments, such as the nerve terminal. αSyn concentrates at synapses, which have high-energy requirements (Rangaraju et al. 2014; Pathak et al. 2015), suggesting that any effects of αsyn KO on bioenergetics would be most prominent in this compartment. To understand the effect of αsyn KO on respiration specifically in synapses, we examined the respiration of synaptosomes isolated from the brains of 6-month-old control and αsyn KO mice. We did not observe differences in the basal or maximal respiration in either cortical or dopaminergic synaptosomes (Fig. 1*E, F*), although there was a small trend for decreased respiration in the αsyn KO dopaminergic synaptosomes.

**Figure 2.**
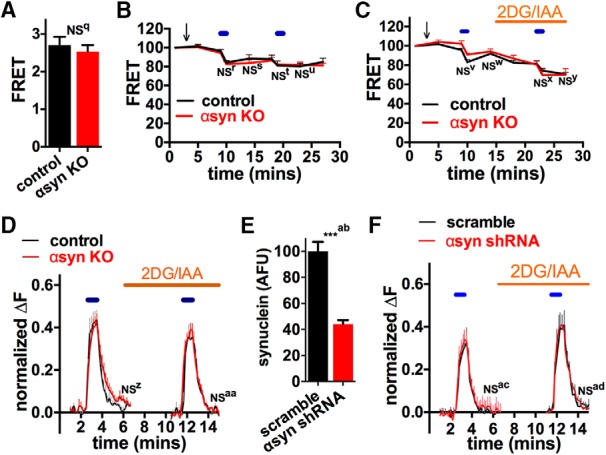
Loss of αsyn does not affect mitochondrial-derived ATP levels at the nerve terminal. ***A–C***, ATP levels of hippocampal neurons were assessed using an ATP YEMK FRET sensor, and synaptic boutons were identified with mCherry-synaptophysin. Basal ATP levels in Tyrodes buffer containing glucose and pyruvate were identical in neurons isolated from control and αsyn KO mice (***A***; *n* = 14–15 coverslips, not significant (NS) by unpaired two-tailed *t* test). Electrical field stimulation (10 Hz*60 s, blue lines) in pyruvate buffer without (***B***) and with (***C***) 2-deoxyglucose (2DG, 5 mM) and iodoacetate (IAA, 1 mM) to completely block glycolysis reduced ATP levels similarly in neurons in control and αsyn KO mice (compilation of two experiments, *n* = 6–7 coverslips/group with 15–20 boutons/coverslip). NS for ATP level of αsyn KO versus control groups at corresponding time points. Note that overall ATP levels (control and αsyn KO) decreased after the first electrical stimulation (***B*** and ***C***, p < 0.01 for ATP at 10 min versus 9 min pre-stimulation time points), while the acute drop in ATP levels after the second stimulations did not reach significance. ***D–F***, Synaptic transmission at individual boutons was assessed using a pH-sensitive GFP targeted to synaptic vesicles (VGLUT1-pHluorin), again in pyruvate buffer, as well as 2DG and IAA to force reliance on glycolysis. Neither αsyn KO (***D***) or shRNA against αsyn (***E*, *F***) affected synaptic vesicle cycling after repeated stimulation (10 Hz*60 s, blue lines). Bar graph confirms that shRNA decreased αsyn levels by immunofluorescence (***E***) (compilation of three experiments, *n* = 10–12 coverslips/group with 10–15 cells/coverslip). NS for extent of endocytosis [(amplitude endocytosis)/(amplitude exocytosis)] versus respective control by two-way ANOVA and Sidak’s posthoc test. All graphs show mean ± SEM.

We next examined how αsyn KO impacted ATP levels at the synapse, which reflect the balance between ATP production and consumption. αSyn KO and control neurons from postnatal hippocampi were co-transfected with ATP FRET sensors (Imamura et al. 2009b) and mCherry-synaptophysin to identify synaptic boutons and then cultured for 10 days before imaging. As expected, basal ATP levels were normal with glucose and pyruvate treatment in αsyn KO neurons (Fig. 2*A*). To specifically examine the capacity of αsyn KO mitochondria to produce ATP, we forced neurons to rely on mitochondria-derived ATP by acutely blocking glycolysis (switching to no-glucose media with glycolytic inhibitors). Under these conditions, the assay sensitively detects decreases in energy caused by either acute pharmacologic or chronic, genetic mitochondrial deficits. Specifically, each comparison was sensitive to an ≈10%–20% decrease in ATP FRET signal with 90% power and an alpha of 0.05 (statistical table), while the FRET must decrease by ≈40% from baseline to pass the threshold level (corresponding to ≈0.8 mM ATP) required for endocytosis (Pathak et al. 2015; Shields et al. 2015). Furthermore, because bioenergetic deficits may only appear when energy requirements increase (Pathak et al. 2015; Shields et al. 2015), we tested if αsyn KO influences the ability of synapses to maintain ATP levels when their energy requirements are increased (Attwell and Laughlin, 2001). Even when neural activity was augmented with electrical field stimulation (10 Hz*60 s) (Nemani et al. 2010), ATP levels decreased similarly with or without αsyn (Fig. 2*B*, *C*).

We also assessed whether loss of αsyn affected synaptic vesicle cycling (particularly endocytosis), an ATP-consuming process that is sensitive to decreases in ATP (Rangaraju et al. 2014; Pathak et al. 2015; Shields et al. 2015). To monitor synaptic vesicle cycling in individual boutons, we used the VGLUT1-pHluorin reporter, which targets a pH-sensitive GFP to the lumen of synaptic vesicles. The pHluorin does not fluoresce in acidified vesicles, but does when synaptic vesicles fuse and expose their contents to the alkaline extracellular environment (Voglmaier et al. 2006; Nemani et al. 2010). Hippocampal neurons expressing VGLUT1-pHluorin were incubated in buffer with 10 mM pyruvate, but without glucose to favor reliance on mitochondria for energy. Even with glycolytic inhibitors, synaptic vesicle cycling (10 Hz*60 s, which preferentially targets the recycling pool) was normal in synaptic boutons lacking αsyn, further supporting that mitochondrial-derived ATP persists at functionally significant levels (Fig. 2*D*). Because developmental compensation may occur in αsyn KO neurons, we also examined how knocking down αsyn with shRNA impacts ATP levels in rat neurons. The shRNA reduced αsyn expression by ∼60% based on immunofluorescence (Fig. 2*E*); however, this decrease did not affect synaptic vesicle cycling (Voglmaier et al. 2006; Nemani et al. 2010) (Fig. 2*F*).

**Figure 3.**
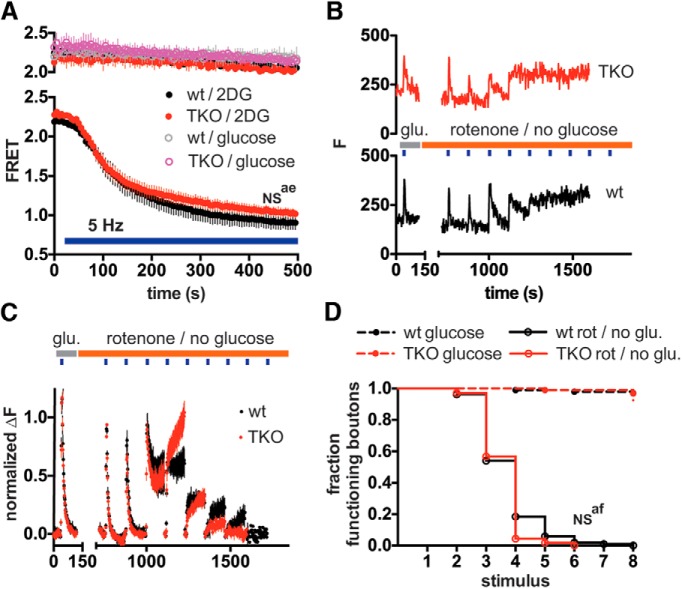
Loss of all three (α, β and γ) syn isoforms does not affect mitochondria-derived ATP or activity-dependent ATP consumption at the nerve terminal. (***A***) ATP levels of syn TKO and control hippocampal neurons expressing the ATP FRET sensor were assessed in normal Tyrode’s buffer with either glucose (30 mM) or 2DG (30 mM) without glucose. Neurons were imaged with or without electrical field stimulation (5 Hz) as indicated. Stimulation with 5 Hz for 475 s in 2DG decreased the FRET signal similarly in wt and TKO neurons (2-way ANOVA, interaction p > 0.99) Data are plotted as mean ± SEM by coverslip. *n* = 4 (wt) and 5 (TKO) coverslips for 5 Hz stimulus/2DG, and 2 coverslips (wt and TKO) for non-stimulated glucose and 2DG controls (50 boutons per coverslip) (***B–D***) Neurons expressing VGLUT1-pHluorin-mCherry were perfused in Tyrodes containing 30 mM glucose (without pyruvate) and stimulated at 30 Hz for 5 s. After continued perfusion for 5 min in either glucose or in 2 μM rotenone without glucose, neurons were stimulated with repeated 5 s 30 Hz bursts every 120 s (blue boxes). (***B***) Sample fluorescence traces from single representative VGLUT1-pHluorin boutons in wild-type (lower) and syn TKO (upper) neurons in rotenone, (***C***) Average fluorescence responses. Data were normalized to the second stimulus response, and points represent mean values by coverslip ± SEM. *n* = 7 (wt) and 8 (TKO) coverslips (18-50 boutons per coverslip) for pyruvate/rotenone experiments, from two independent experiments. (***D***) Fluorescence traces from individual boutons (as in (A)) were scored with regard to synaptic vesicle cycling response at each stimulus burst. The stimulus burst at which the response "failed" was recorded, and data were plotted as survival curves. Boutons were scored as failed if stim ΔF was <10% of the ΔF from first stim, or if ΔF 120s after stimulus was >33% of the peak Fstim-F0 value (ie endocytic failure). Wt and syn TKO boutons in rotenone without glucose progressively failed to respond at a similar rate (p = 0.21 by Gehan-Breslow-Wilcoxon test). *n* = 325 (wt) and 340 (TKO) boutons for rotenone/no glucose, 98 (wt) and 93 (TKO) boutons for glucose-containing Tyrode’s. The average lifespan of boutons by coverslip in rotenone/no glucose was also similar (wt = 3.75 ± 0.253 and syn TKO = 3.61 ± 0.137).

Increased βsyn can have similar albeit less potent detrimental effects to αsyn on mitochondrial functions (Kamp et al. 2010; Nakamura et al. 2011), and loss of all three (α, β and γ, syn TKO) isoforms (but not loss of αsyn alone) decreases mouse lifespan, raising the possibility that β and γsyn compensate to maintain mitochondrial function when αsyn is lost. However, sustained electrical stimulation (5 Hz*475s) decreased ATP levels similarly in syn TKO and control synaptic boutons, suggesting that concurrent loss of isoforms does not affect mitochondria-derived ATP levels at the nerve terminal (Fig. *3A*). As ATP levels depend on the balance between energy production (aerobic respiration and glycolysis) and consumption, we also assessed if syn TKO might impact the rate of ATP consumption. However, when energy production was blocked (the respiratory chain was blocked with rotenone (2 μM), and external glucose was removed to limit glycolysis), and energy consumption was increased with repetitive electrical stimulation (30 Hz*5s every 120s), the stimulus-dependent endo- and exocytic response of syn TKO and control boutons failed at the same rate (Fig. *3B-D*), indicating that ATP levels fell below the threshold level needed to support synaptic vesicle cycling at the same rate (Pathak et al. 2015; Shields et al. 2015). Since the ATP level reflects a balance between ATP production and consumption, these results suggest that the net balance of ATP consumption and any residual ATP production by glycolysis is also similar in syn TKO and control synaptic boutons.

### Lowering αsyn Does Not Impact Mitochondrial Distribution in Axons

**Figure 4.**
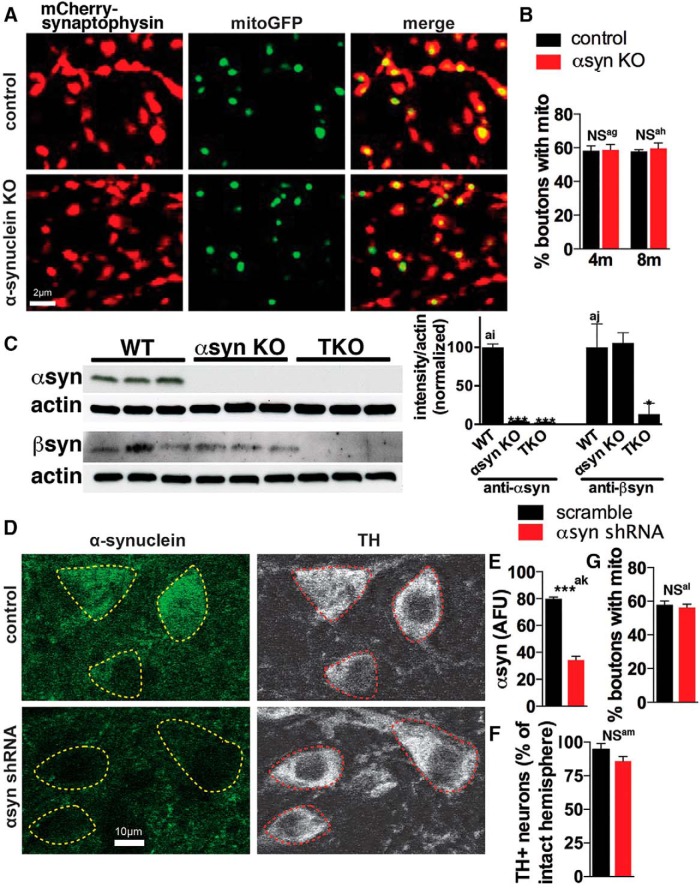
Loss of αsyn does not affect the distribution of mitochondria in axons of nigrostriatal DA neurons *in vivo*. ***A–B***, Adeno-associated viruses (AAVs) expressing mitochondria-targeted GFP (mitoGFP; green, to visualize mitochondria) and mCherry-Synaptophysin (red, to visualize synaptic boutons) in DIO constructs (Sohal et al. 2009) that express only in Cre-expressing neurons were coinjected into the substantia nigra pars compacta (SNc) of 3- and 7-month-old DATcre control and αsyn KO-DATcre mice. Mice were sacrificed one month later at 4 and 8 months of age, respectively. Roughly 60% of control and αsyn KO synaptic boutons show mitochondria in the caudate putamen (CPu) (*n* = 3–4 mice per group, where each value is the mean of 18–21 fields; NS = not significant by two-way ANOVA and Sidak’s posthoc test). ***C***, Western blot shows that αsyn KO mice have similar levels of βsyn as controls (*n* = 3 mice per group; *p < 0.05, ***p < 0.001 by one-way ANOVA and Dunnet’s posthoc test). ***D–G***, AAVs expressing expressing mitoGFP and mCherry-Synaptophysin in DIO constructs were co-injected with shRNA scramble TagBFP or shRNA αsyn TagBFP into the SNc of 7-month-old Dat^icre/wt^ mice, and brains were harvested 6 weeks later. ***D–E*,** shRNA against αsyn decreased αsyn immunofluorescence ∼60% versus shRNA scramble in DA neurons (*n* = 3–4 mice, 57–86 cells per mouse (αsyn immunofluorescence level of individual cells for each mouse is shown in Fig. 4E-1); *p < 0.001 by unpaired two-tailed *t* test), identified by tyrosine hydroxylase (TH), but had no effect on either the number of TH+ neurons as measured by stereology (***F***; *n* = 6-8 mice per group; NS = not significant (p = 0.16) by unpaired two-tailed *t* test) or on the localization of mitochondria to synaptic boutons (***G***; *n* = 3–4 mice per group, where each value is the mean of 6–10 fields; NS = not significant by unpaired two-tailed *t* test). All graphs show mean ± SEM.

Even when the intrinsic function of individual mitochondria is normal, changes in the distribution of mitochondria could create regions within neurons (especially axons) without sufficient mitochondria to meet energy requirements, leading to energy failure in that region. αSyn primarily locates to presynaptic terminals (Jakes et al. 1994; Iwai et al. 1995), and mutant A53T αsyn decreases the movement and density of mitochondria in axons (Choubey et al. 2011; Li et al. 2013). To determine if endogenous αsyn influences the localization of axonal mitochondria to synapses, we used cre-dependent AAV-based viral reporters to visualize mitochondria [mitochondrial-targeted GFP (mitoGFP)] specifically in individual DA neurons and their synapses (mCherry-synaptophysin) (Berthet et al. 2014) in DATcre control and αsyn KO-DATcre mice that express Cre recombinase selectively in *Slc6a3* (dopamine transporter, DAT)-expressing DA neurons (Backman et al. 2006). αSyn KO did not affect the proportion of DA boutons containing mitochondria (∼60%) in the caudate putamen (CPu) (Fig. 4*A*, *B*).

10.1523/ENEURO.0216-16.2017.f4e-1 Figure 4E-1 Source data for Figure 4E. Scatter graphs show the relative αsyn immunofluorescence levels of individual DA neurons from mice treated with αsyn and scramble shRNA. n=3–4 mice, 57–86 cells per mouse. Download Figure 4E-1, TIF file.

Notably, αsyn KO mice may have developmental changes that compensate for the loss of αsyn (Kuhn et al. 2007). Although we did not detect changes in the level of βsyn in total brain lysates (Fig. 4*C*), other groups found it upregulated in the midbrain of αsyn KO mice (Robertson et al. 2004; Thomas et al. 2011) and that compensation could occur independent of changes in expression. To further exclude the possibility of any developmental compensation, we also examined if lowering αsyn with shRNA in adult mice (Gorbatyuk et al. 2010; Kanaan and Manfredsson, 2012) would impact the axonal localization of mitochondria. Using AAV expressing an shRNA against αsyn, we lowered αsyn levels in DA neurons by ∼60% (Figs 4*D*-*E*, 4E-1) (as measured by immunofluorescence), a level of decrease that may not be quite sufficient to produce neuronal loss in rats (Gorbatyuk et al. 2010), and mice may also be more resistant (Benskey et al. 2016b). Consistent with this, there was no significant loss of DA neurons in the αsyn shRNA group (Fig. 4*F*), although there was a trend for decreased TH+ counts. This level of αsyn decrease also did not affect the proportion of boutons containing mitochondria (Fig. 4*G*). Therefore, αsyn levels can be significantly lowered in DA neurons without impacting the synaptic targeting of mitochondria. We cannot exclude the possibility that further lowering of αsyn by shRNA such that the DA neurons die would have disrupted mitochondria, and it would be difficult to attribute such a change specifically to αsyn lowering as mitochondria are typically disrupted during neuronal death, regardless of the cause. Nonetheless, when considered with the lack of effect in αsyn KO mice, our data suggest that either αsyn does not normally impact mitochondrial distribution in DA axons or that other factors compensate for αsyn loss (Nakamura et al. 2011).

**Figure 5.**
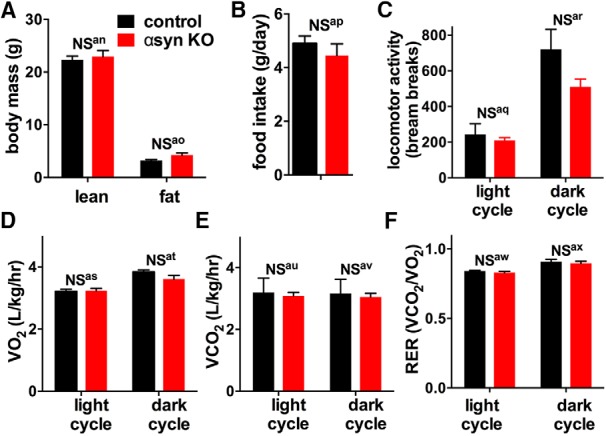
αsyn KO does not impact total body metabolism in mice. ***A***, Body composition was measured using EchoMRI. Control and αsyn KO had identical lean and fat body mass composition at 6 months of age. ***B–F***, Body metabolism was assessed using a Comprehensive Lab Animal Monitoring System (CLAMS; Columbus Instruments). αsyn KO and control mice had similar daily food intake (***B***) and locomotor activity (***C***). They also had similar Vo_2_ (***D***), Vco_2_ (***E***), and respiratory exchange ratio (RER, ratio of Vco_2_ produced and Vo_2_ used) (***F***) during both the light and dark cycles. *n* = 6 mice per group; NS = not significant by two-way ANOVA and Sidak’s posthoc test. All graphs show mean ± SEM.

αSyn is expressed throughout the brain, including in regions that influence respiration and metabolism. αSyn is also present at high levels in certain peripheral tissues including in red blood cells, liver and spleen (Kuo and Nussbaum, 2015), and phosphorylated αsyn accumulates in peripheral tissues in PD (Beach et al. 2010). However, there is very little information on if αsyn expression in these areas impacts metabolic functions. Because αsyn-lowering therapies will likely lower αsyn levels throughout the brain and in peripheral tissues, we also examined if interactions between αsyn and mitochondria affect energy metabolism on a whole-body level using metabolic-cage analyses and CLAMS (Columbus Instruments). At 6 months of age, αsyn KO mice had total and lean body masses similar to controls, as assessed by EchoMRI (Fig. 5*A*). Food consumption (Fig. 5*B*) and total locomotor activity (Fig. 5C) were also unchanged, although the assessment of activity lacked sensitivity due to high variability, and there was a trend toward less movement in the dark cycle in the αsyn KO group. However, importantly, αsyn KO did not affect oxygen consumption (VO_2_), carbon dioxide production (VCO_2_), or the respiratory-exchange ratio (RER; [dot]Vco_2_/VO_2_) in either the light or dark cycle (Fig 5*D*–*F*). Taken together, these data strongly suggest that loss of αsyn does not impact total energy consumption in mice.

## Discussion

αSyn likely plays a central role in the pathogenesis of sporadic PD. Mitochondria are also compromised in PD, so therapeutic lowering of αsyn will be done in the context of damaged mitochondria. In addition, increased αsyn disrupts a range of mitochondrial functions, suggesting that decreasing αsyn also influences mitochondrial function. However, here, we show that loss of αsyn does not significantly impact the intrinsic bioenergetic function of mitochondria (i.e., respiration and ATP levels) in rodent neurons, even regionally at the synapse where αsyn concentrates. Loss of αsyn also fails to influence the localization of mitochondria in DA axons or disrupt normal energy consumption in the whole body. Thus, our findings suggest that either αsyn has no significant physiologic impact on mitochondrial bioenergetic function, or that any such functions are fully compensated for when lost or emerge only in the presence of specific stressors.

Increased αsyn expression selectively inhibits complex I function (Devi et al. 2008; Liu et al. 2009; Chinta et al. 2010; Loeb et al. 2010) or the flux between complex I and III (Ellis et al. 2005; Devi et al. 2008). However, we do not yet understand the precise mechanisms of these effects or if the decrease in complex I function impacts energy production. Insufficient energy could also result from changes in the mass or distribution of mitochondria, even if the mitochondria have normal function. Indeed, increased mutant A53T αsyn augments Parkin-dependent mitophagy in cortical neurons (Choubey et al. 2011) and the number of mitochondria in autophagosomes in midbrain DA neurons (Chinta et al. 2010). However, we found that decreased αsyn did not affect the bioenergetic function of mitochondria, including regionally at the synapse, or the mass or distribution of mitochondria in nigrostriatal DA neurons *in vivo*.

The lack of effect of αsynKO on bioenergetic function has three potential explanations. The first is that αsyn KO does actually impair bioenergetic function but our studies failed to detect this due to insufficient sensitivity. Indeed, many of our assays lacked the sensitivity to reliably detect changes less than ≈10% - 15%, and hence, subtle changes would not have been detected. However, all of the approaches used to assess bioenergetic function have been validated for their sensitivity to detect the effects of acute pharmacologic and chronic genetic inhibitors of respiration, and the uniform lack of significant changes across multiple complementary approaches provides strong evidence that αsyn KO does not significantly impact bioenergetic function in the paradigms studied. Insufficient sensitivity could also have resulted if we assayed the wrong type of cell or the wrong subcellular compartment. In particular, our study focuses on neurons because αsyn primarily localizes to neurons normally and accumulates in neurons in PD. Moreover, within neurons, we focused on synapses where most αsyn KO localizes. To specifically assay nigrostriatal DA neurons we examine DA synaptosomes. However, recognizing the potential for artifact from the antibody bead-based isolation of DA synaptosomes, we also examined cortical synaposomes that were isolated without use of antibodies. Since synaptosomes likely have distinct bioenergetic properties from intact neurons, and even the standard isolation of cortical synaptosomes may introduce artifacts, we assayed mitochondria-derived ATP levels in individual synaptic boutons of live neurons in complementary assays.

Insufficient sensitivity for an effect of decreasing αsyn on bioenergetics could also have resulted if decreasing αsyn affects only certain neuron types that we failed to assay. For instance, although we assayed DA neurons when possible, many of our assays focused on hippocampal neurons. However, we hypothesize lowering αsyn will have similar effects across most neuron types. Indeed, αsyn is present in neurons throughout the brain and, undoubtedly, has normal functions outside of nigrostriatal DA neurons. (Bendor et al. 2013; Benskey et al. 2016b) Thus, we must understand the impact of lowering αsyn on nonDA neurons, in addition to DA neurons, since any αsyn lowering therapy will likely be delivered to the entire brain. Moreover, the fact that human nigrostriatal DA neurons are susceptible to increased αsyn in familial forms of PD does not mean they will also be more susceptible to αsyn loss. In fact, it could mean just the opposite, and the underlying mechanisms of toxicity of increasing versus decreasing αsyn could also be very different. For instance, increased αsyn in some forms of PD may cause toxicity through a toxic gain of function of αsyn, but loss of αsyn could produce toxicity through loss of its normal function. Moreover, in PD, αsyn accumulates in many nonDA neurons, including hippocampal neurons (e.g., see Hall et al. Brain, 2014), presumably contributing to the many non-motor features of the disease. As such, hippocampal neurons are an important and appropriate system to study normal αsyn biology, and the wealth of preexisting studies in this neuron type facilitates interpretation of our results. However, the possibility remains that αsyn-lowering compromises bioenergetics only in certain other neuron types.

A second possibility for a lack of effect of decreasing αsyn on bioenergetic function is that αsyn may normally interact with mitochondria and influence respiration, but the effects of αsyn loss may be compensated for by other factors, such as βsyn, which has similar, albeit less, potent effects on mitochondrial morphology when overexpressed (Nakamura et al. 2011; Taschenberger et al. 2013). Indeed, the three syn isoforms can compensate for each other, because αsyn, βsyn, and γsyn single–KO mice and αsyn/βsyn double–KO mice have normal lifespans (Chandra et al. 2004), but triple syn–KO mice die early (Greten-Harrison et al. 2010). As evidence against this possibility, however, we found no effect of synTKO on mitochondria-derived ATP levels at the nerve terminal or on the rate of ATP consumption. In apparent contradiction, Ludtmann et al. (Ludtmann et al. 2016) recently reported that synTKO neurons have impaired bioenergetic function, suggesting that other syn isoforms compensate for certain αsyn-effects on mitochondria. The reasons for this discrepancy are unclear, but could reflect differences between subcellular compartments. Specifically, our studies on synTKO neurons focused on changes at the nerve terminal where αsyn and βsyn accumulate. They also observed ATP changes using a mitochondria-targeted ATP sensor, while we examined cytosolic ATP levels, raising the possibility that synuclein isoforms might specifically alter ATP levels in the mitochondria. However, other methodological differences including the use of permeabilized versus intact neurons and the use of Mg^2+^ homeostasis (versus our use of synaptic vesicle cycling) to assay for ATP consumption may also underlie some of the differences, and will require additional experimentation to resolve.

A third possibility is that αsyn normally has minimal interactions with mitochondria and little effect on bioenergetic function. Although increased αsyn disrupts mitochondrial morphology and function, these effects may reflect direct toxicity from the interaction of αsyn oligomers with mitochondria (van Rooijen et al. 2009; Nakamura et al. 2011; Nakamura, 2013; Luth et al. 2014), and they may not occur under normal conditions. Also, while endogenous αsyn can influence mitochondrial morphology (Kamp et al. 2010; Norris et al. 2015), and disrupt mitochondrial protein import (Di Maio et al. 2016), these changes may not be sufficiently robust to compromise respiration under basal conditions, although may be more prominent under pathologic conditions as in PD. Furthermore, although αsyn KO mice resist MPTP and other mitochondrial toxins (Dauer et al. 2002; Klivenyi et al. 2006), and decreasing endogenous αsyn levels protects against rotenone (Zharikov et al. 2015), the mechanism of these effects may be independent of bioenergetic function and the other parameters studied here.

Importantly, our study does not exclude the notion that lowering αsyn may impact other mitochondrial functions, such as mitochondrial Ca^2+^ import and buffering (Cali et al. 2012; Guardia-Laguarta et al. 2014), reactive oxygen species production, and lipid metabolism (Ellis et al. 2005; Cole et al. 2008; Nakamura et al. 2008; Nunnari and Suomalainen, 2012). Nonetheless, significant changes in any of these parameters would likely affect bioenergetic function and mitochondrial morphology, suggesting that any such changes would likely be subtle.

Our findings show that αsyn can be safely lowered in mice without affecting mitochondrial bioenergetics. We believe that these studies suggest that therapeutically lowering αsyn is unlikely to further disrupt mitochondrial bioenergetic function in PD. These results will need to be established in humans, especially if intended for therapies for PD patients, which will require many years. Moreover, lowering αsyn could also produce toxicity through non-mitochondrial functions, such as disrupting synaptic vesicle release. Considering the rapid development of αsyn-lowering therapies, we can expect to gain new insights into the safety and biological impact of αsyn-lowering therapies over the coming decade.

## Synthesis

Reviewing Editor: Julie Andersen, Buck Institute Decisions are customarily a result of the Reviewing Editor and the peer reviewers coming together and discussing their recommendations until a consensus is reached. When revisions are invited, a fact-based synthesis statement explaining their decision and outlining what is needed to prepare a revision will be listed below. The following reviewer(s) agreed to reveal their identity: Edward Burton, Laura Volpicelli-Daley.

The reviewers appreciated the additional changes made in response to previous comments. Additional experiments have however been requested by Reviewer #2 as well as a further discussion around the statistical power of the studies. The manuscript could be further improved with a more nuanced discussion of possible caveats as pointed out by the reviewers.

Reviewer #1

The authors have made extensive changes to address the reviews, including critical discussion of the power of the experiments to detect changes and better framing of the study in the context of recent literature.

I disagree about the use of the term “Parkinson's disease” to indicate anything other than sporadic Lewy body disease with a typical clinical presentation. I will defer to the editor about the (in my opinion incorrect) use of “genetic PD”, which is - as the authors point out - unfortunately fairly widespread.

The authors may have misunderstood my point about the Di Maio paper, which I understood to show that a-syn interaction with TOM20 disappears after reduction of cellular α-synuclein, and that the asyn/TOM20 interaction inhibits import of complex I subunits. Presumably this is not sufficiently robust in the experimental systems used here to result in an abnormality of cellular bioenergetics.

Reviewer #2

In this manuscript, the authors examine the impact of absence of alpha-synuclein on mitochondrial function. This data is critical given the potential role of alpha-synuclein in mitochondrial function and that current therapies are being developed for Parkinson's disease that attempt to reduce alpha-synuclein levels. I would however, like to see the following:

1. It is clear that absence of alpha-synuclein does not cause a major impact on OCR compared to the pharmacological compounds that increase or reduce OCR. However, because these are neurodegenerative diseases, it is possible that a small, even 10% decrease in OCR could be important with respect to long term treatment. The study was underpowered to detect small significant differences . Therefore, I suggest toning down their claims in the title and abstract such as “minimal effects of alpha-synuclein on mitochondrial bioenergetics,” and that “absence of alpha-synuclein causes a small, but not statistically significant decrease in OCR”, instead of saying absence of alpha-synuclein “does not affect respiration”.

2. I would really like to see the effect of knocking down alpha-synuclein on OCR in the primary hippocampal neurons. Alpha-synuclein is only expressed in mature synapses. So it is possible that allowing alpha-synuclein to be expressed, then reducing its levels could have a major impact on mitochondrial respiration or neuronal function. This would also be more relevant to therapeutic potential of reducing alpha-synuclein than the knockout neurons.

3. For the knockdown experiments, I would like to see western blots of alpha-synuclein with quantitation. I would also like to see the immunofluorescent images of the alpha-synuclein knockdown with images captured with the same settings. The bar graphs showing quantitation of immunofluorescence is not sufficient.

4. Can the authors provide the statistics on the treatments in figure 2? Did stimulation or inhibition of glycolysis significantly change ATP levels as measured by the ATP YEMK FRET sensor?

5. In figure 3B, is the enhanced F in the TKO because of impaired synaptic vesicle endo/exocytosis as shown previously (PMID: 25009269)? It is worth pointing this out.

6. The authors could discuss the DiMaio study a little more. The DiMao STM paper really showed that abnormal alpha-synuclein (oligomers, phospho-synuclein, dopamine modified synuclein) inhibits mitochondrial protein import. Therefore, this current study combined with the DiMaio study suggest that the abnormal alpha-synuclein is toxic in disease, whereas normally, alpha-synuclein probably does not play a major role in mitochondrial function.
