# Supplementary figures and images for: Loss of α-Synuclein Does Not Affect Mitochondrial Bioenergetics in Rodent Neurons
Source: eNeuro. 2017 Apr 28;4(2):ENEURO.0216-16.2017. doi: 10.1523/ENEURO.0216-16.2017 (PMC5409983; doi:10.1523/ENEURO.0216-16.2017)

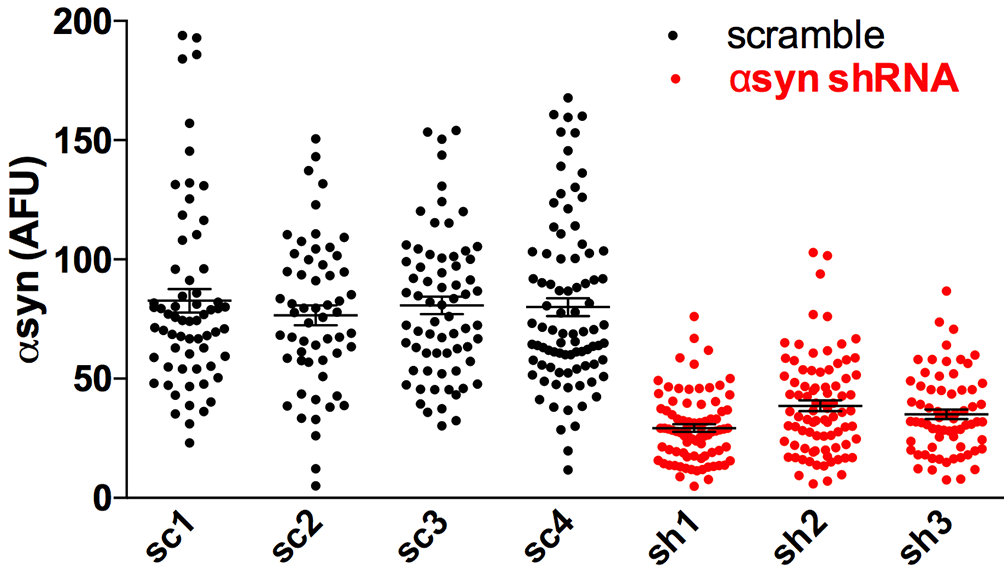

Supplement: Figure 4E-1 [file enu002172295so6.tif]
